# A comparative analysis of blood pressure categorization using 24-hour ambulatory blood pressure monitoring in young women with presumed chronic hypertension with and without a history of pregnancy

**DOI:** 10.1016/j.xagr.2026.100616

**Published:** 2026-02-12

**Authors:** Srishti Gupta, Megan R. Knutson Sinaise, Jennifer Zaborek, Kara K. Hoppe

**Affiliations:** a Department of Obstetrics and Gynecology, School of Medicine and Public Health, University of Wisconsin–Madison, Madison, WI (Gupta, Knutson Sinaise, and Hoppe); b Department of Biostatistics and Medical Informatics, University of Wisconsin–Madison, Madison, WI (Zaborek).

**Keywords:** chronic hypertension, 24-hour ambulatory blood pressure, hypertensive disorders of pregnancy, young adults

## Abstract

**BACKGROUND:**

Hypertension among childbearing-age women is often underrecognized, undertreated, and when diagnosed, misclassified. Accurate diagnosis of hypertension is imperative, especially in cases of pregnancy because it directly influences medical management, delivery plan, and treatment decisions.

**OBJECTIVE:**

This study aimed to assess the 24-hour ambulatory blood pressure monitoring results of women from the MyHEART trial who had a presumed clinical diagnosis of chronic hypertension, and to determine the incidence of masked, white coat, nocturnal, and confirmed hypertension. Secondarily, we also assessed self-reported pregnancy history and the impact of having a prior pregnancy with or without hypertension on their 24-hour ambulatory blood pressure monitoring results.

**STUDY DESIGN:**

We conducted a secondary analysis of the data from 221 women who completed 24-hour ambulatory blood pressure monitoring during a preenrollment visit to confirm eligibility for the MyHEART trial. All women, regardless of whether they enrolled in the trial, were interviewed via phone about their history of pregnancy, hypertensive disorders of pregnancy, and/or antihypertensive medication use. Statistical analysis was performed using R software and Mann–Whitney–Wilcoxon and chi-square tests.

**RESULTS:**

Among 221 women, 142 (64.3%) had confirmed chronic hypertension, 73 (33.0%) had white coat hypertension, 56 (25.3%) had nocturnal hypertension, and 43 (19.5%) had masked hypertension according to 24-hour ambulatory blood pressure monitoring results. The incidence of confirmed chronic, white coat, masked, or nocturnal hypertension in the 55 women with a history of pregnancy did not differ significantly compared with women without a history of pregnancy. Additionally, 47 of the 55 women reported hypertension during pregnancy, but their ambulatory blood pressure monitoring results were not affected by the specific hypertensive diagnosis assigned during pregnancy. Within this subgroup, the ambulatory blood pressure monitoring results varied by race. On average, Black women had significantly higher ambulatory systolic blood pressure readings and an increased incidence of nocturnal hypertension compared with their White counterparts.

**CONCLUSION:**

The integration of diagnostic tools such as 24-hour ambulatory blood pressure monitoring or, alternatively, home blood pressure monitoring for women with elevated blood pressures—particularly those of childbearing age—allows for more precise classification of hypertension. Accurate diagnosis supports optimal medical management and treatment decision-making, including during pregnancy, and helps avoid unnecessary interventions in individuals with white coat hypertension. Ultimately, improved diagnostic clarity enhances our ability to deliver tailored, evidence-based care by providing a more nuanced understanding of each patient’s blood-pressure profile.

**AJOG Global Reports at a Glance**

**Why was this study conducted?**

This study aimed to evaluate the prevalence of white coat, masked, and nocturnal hypertension confirmed using 24-hour ambulatory blood pressure monitoring in young adult women with a clinical diagnosis of chronic hypertension with and without a history of pregnancy.

**Key findings**

White coat hypertension was identified in 33% of women in the overall study population and 34.5% of those with a history of pregnancy. Black women with prior pregnancies had higher ambulatory systolic blood pressure and a greater incidence of nocturnal hypertension.

**What does this add to what is known?**

This study demonstrates the benefit of using 24-hour ambulatory blood pressure monitoring to properly categorize high blood pressure and identify white coat, nocturnal, and masked hypertension in young adult women with a clinical diagnosis of chronic hypertension. These findings highlight the need for improved cardiovascular surveillance and proper categorization of hypertension type to guide hypertension diagnosis and management in women of childbearing age.

## Introduction

Hypertension (HTN) is a life-threatening condition that affects women in all stages of life and significantly increases their risk of developing cardiovascular disease, diabetes, chronic kidney disease, and other morbidities. Despite being a preventable and modifiable risk factor, it still accounts for 1 in 5 deaths among women in the United States.^1^ Among women of reproductive age, HTN carries particular significance because the prevalence of chronic HTN during pregnancy in the United States has doubled between 2007 and 2021.^2^ Furthermore, hypertensive disorders of pregnancy—including chronic HTN, gestational HTN, preeclampsia, eclampsia, and chronic HTN with superimposed preeclampsia—remain a significant cause of perinatal mortality and morbidity, accounting for 10% of all maternal deaths.^3^

In nonpregnant individuals, stage 1 HTN is defined as having blood pressure (BP) readings of ≥130/80 mm Hg.^4^ Notably, the MyHEART trial, which commenced in October 2017 before the release of the updated 2017 American Heart Association (AHA)/American College of Cardiology (ACC) HTN guidelines, defined chronic HTN as BP exceeding 140/90 mm Hg.^5^ During pregnancy, chronic HTN is defined as persistently elevated BP levels of ≥140/90 mm Hg, either before conception or before the 20th week of gestation.^6^ Beyond chronic HTN, elevated BP can manifest in several distinct patterns, which can be classified into different HTN subtypes, including white coat, masked, daytime, and nocturnal HTN. White coat HTN is defined as having elevated BP measurements in the clinical setting (eg, a doctor’s office or hospital) but normal BP outside the clinical setting due to transient stress or anxiety related to being in the clinical environment.^7^^,^^8^ According to the AHA, white coat HTN affects approximately 25% of the general nonpregnant adult population, but its prevalence in pregnancy is uncertain, ranging from 4% to 30%.^9^ Masked HTN, conversely, is defined as having normal BP measurements in a clinical setting but elevated BPs outside the clinic (ambulatory or home BP).^7^ It has been reported in 15% to 30% of patients who are normotensive in clinic, but its prevalence and clinical implications during pregnancy and the postpartum period remain poorly understood.^9^^,^^10^ Presently, nocturnal HTN is defined as nighttime BP measurements ≥110/65 mm Hg and can occur among women with normal office or daytime BP.^4^ As such, despite being associated with adverse cardiovascular events and organ damage, it frequently remains undiagnosed in standard clinical practice.^11^^,^^12^ Accurate identification of these BP subtypes is essential because they are associated with differing cardiovascular risk profiles and require distinct monitoring and management strategies. Inability to conduct accurate categorization can also worsen health outcomes because it may lead to unnecessary treatment intensification or, conversely, to inadequate monitoring and management.^13^ In the context of pregnancy, this significantly increases the risk of complications and can lead to adverse maternal and neonatal outcomes.

This presents a diagnostic challenge in routine clinical practice. Office BP measurements remain the main method used to diagnose HTN, including during pregnancy, but reliance on this method alone fails to identify clinically important HTN subtypes.^14^ Ambulatory blood pressure monitoring (ABPM) is considered the gold standard for diagnosing HTN in individuals with elevated clinic BP.^15^ Both the AHA and American College of Obstetricians and Gynecologists (ACOG) endorse the use of 24-hour ABPM to identify white coat HTN during pregnancy.^9^^,^^16^ However, its use in clinical practice remains limited. Moreover, despite being shown to be a better predictor than conventional BP readings for identifying HTN, few studies have evaluated its utility specifically in women of childbearing age. Accordingly, this study was conducted to evaluate whether ABPM conducted outside of pregnancy provides more accurate HTN classification in women of childbearing age, and to assess how such classification can better inform pregnancy-related risk assessment and management.

MyHEART is a trial that was designed to assess whether telephone-based health coaching could improve BP control in young adults with uncontrolled HTN. ABPM testing was used at enrollment, and again at 6- and 12-month follow-up.^5^ We therefore conducted a secondary analysis of the MyHEART trial to explore the utility of ABPM in confirming chronic HTN and accurately classifying HTN control status and type in women with and without a history of pregnancy. The primary aim of this study was to assess whether 24-hour ABPM—including daytime systolic and diastolic BP, nocturnal BP, and final classification (chronic HTN, white coat HTN, masked HTN, nocturnal HTN, or normotension)—was concordant with participants’ preexisting clinical diagnosis of chronic HTN. The secondary aim was to evaluate these same ABPM findings among women with a self-reported pregnancy history, further stratified by prior normotensive vs hypertensive pregnancy.

## Materials and methods

This is a secondary analysis of the MyHEART study, a multicenter randomized controlled trial conducted at 2 large Midwestern academic centers, with enrollment from October 2017 through December 2021 (NCT03158051). The study design, inclusion/exclusion criteria, and results have been published.^5^ In brief, the study population included young men and nonpregnant women (aged 18–39 years) who had uncontrolled HTN at enrollment. To identify uncontrolled HTN at enrollment in this study, potential participants who received their medical care in the study institutions were required to have a minimum of 2 HTN-coded office visits (International Statistical Classification of Diseases and Related Health Problems, Tenth Revision [ICD-10]) on different dates within 24 months before eligibility assessment, including at least 1 code in the past 18 months. Two clinic BP measurements determined eligibility for a study invitation (ie, systolic BP ≥140 mm Hg and/or diastolic BP ≥90 mm Hg), with the most recent BP measurement obtained within 90 days. BP measurements obtained in inpatient, emergency department, urgent care, or self-reported settings were excluded.^5^ The 2017 AHA/ACC guidelines were published during the conduct of this trial, lowering the BP threshold for a diagnosis of HTN. However, the diagnostic BP of ≥140 mm Hg for systolic BP and/or ≥90 mm Hg for diastolic BP was maintained throughout the study because this was the standard diagnostic threshold for HTN per US guidelines when the study was initiated. All remaining eligible participants who completed visit 1 received placement of a 24-hour ABPM monitor (OnTrak [90277]; Spacelabs Healthcare, Snoqualmie, WA) to confirm a HTN diagnosis (ie, exclude white coat HTN). Participants were eligible if the mean 24-hour ABPM result was systolic ≥130 mm Hg and/or diastolic ≥80 mm Hg, and/or the mean awake ABPM result was systolic ≥135 mm Hg and/or diastolic ≥85 mm Hg. Participants were ineligible if the 24-hour ABPM demonstrated normotensive findings, white coat HTN (<130/80 mm Hg on 24-hour monitoring), or controlled HTN while receiving antihypertensive medications. We did not explicitly exclude participants for secondary causes of HTN; however, most were presumed to have essential HTN.

For this analysis, we identified all female participants who presented for consideration of enrollment in the MyHEART study, provided consent (IRB#: 2017-0372), and completed the baseline 24-hour ABPM. All female participants—regardless of randomization status, study withdrawal, loss to follow-up, or study completion—were contacted by telephone to participate in a follow-up interview to collect additional data. The interview questions are detailed in the Appendix. According to the responses, participants were stratified by history of pregnancy and history of HTN during pregnancy. Within the overall cohort and each subgroup, ABPM data were used to determine the appropriate BP classification: confirmed chronic HTN (office BP ≥140/90 mm Hg with overall ABPM [combining awake and sleep periods] ≥130/80 mm Hg or mean daytime ABPM ≥135/85 mm Hg) white coat HTN (office BP ≥140/90 mm Hg with ABPM <130/80 mm Hg or awake average <135/85 mm Hg), masked HTN (office BP <140/90 mm Hg with 24-hour ABPM ≥130/80 mm Hg or awake average ABPM ≥135/85 mm Hg), nocturnal HTN (sleep BP average ≥120/80 mm Hg), or normotension (office and ABPM BPs <130/80 mm Hg). These thresholds were based on standard office and ABPM HTN diagnostic criteria at the time of initiation of the MyHEART trial, which preceded the release of the updated 2017 AHA/ACC guidelines.^11^^,^^17^^,^^18^ The different study groups were compared according to history of pregnancy and history of HTN during pregnancy. Statistical analysis was performed using the R software (R Foundation for Statistical Computing, Vienna, Austria). Mann–Whitney–Wilcoxon tests were used to analyze continuous variables and chi-square tests for categorical variables. Variables included education, number of HTN medications, and self-perceived health status. The authors will provide study data upon request by the Editors.

## Results

**Figure 1:**
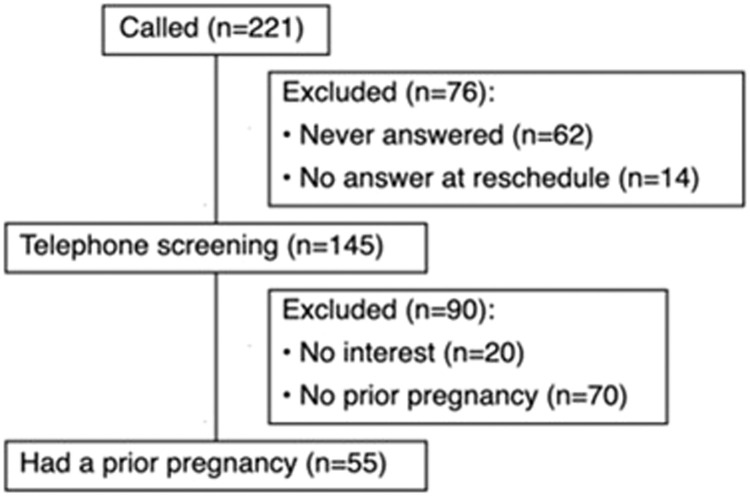
CONSORT flow diagram illustrating participant inclusion process *CONSORT*, Consolidated Standards of Reporting Trials.

Among the participants who presented for consideration of enrollment into the MyHEART trial, 513 completed baseline 24-hour ABPM, and 221 of those were women. To assess eligibility for this study, we attempted to contact all 221 female participants who presented for the MyHEART trial; 76 of could not be reached. Of the 145 women who completed the screening, 20 declined to participate and 70 did not have a history of pregnancy, resulting in a subgroup of 55 women with a history of pregnancy (Figure 1).

Gupta. Ambulatory blood pressure monitoring for blood pressure categorization in young women with presumed chronic hypertension. Am J Obstet Gynecol Glob Rep 2026.

The demographics of all female participants who completed ABPM testing for the MyHEART trial were stratified by exclusion status and pregnancy history, as shown in Table 1.

**Table 1:** Baseline participant characteristics by participation in substudy

Variable	Overall	Excluded	Prior pregnancy	*P* value
Total	221	166	55	
Age	33.3 [19.0–39.0]	32.7 [19.0–39.0]	35.3 [26.0–39.0]	.00
Alcohol beverages/wk	2.6 [0.0–25.0]	2.5 [0.0–25.0]	3.1 [0.0–23.0]	.87
Body mass index (kg/m^2^)	35.3 [19.5–62.2]	35.3 [19.5–62.2]	35.5 [21.1–54.3]	.55
Night dip diastolic ABPM (\%)	16.9 [0.0–37.2]	15.9 [0.0–33.3]	19.3 [0.0–37.2]	.10
Number of children at home	1.2 [0.0–6.0]	0.9 [0.0–6.0]	2.3 [0.0–6.0]	<.001
Office systolic BP (mm Hg)	130.9 [102.0–192.0]	131.2 [102.0–192.0]	130.2 [102.7–175.7]	.65
Office diastolic BP (mm Hg)	88.8 [52.7–131.7]	88.9 [52.7–131.7]	88.4 [57.3–130.3]	.66
Waist circumference (cm)	108.4 [65.0–163.0]	108.6 [65.0–163.0]	107.6 [80.0–145.0]	.84
Weight (kg)	95.7 [45.0–182.0]	95.5 [45.0–182.0]	96.5 [61.0–146.0]	.52
Cigarette tobacco status				.46
I currently smoke cigarettes	27 (12.2)	18 (10.8)	9 (16.4)	
I have never smoked cigarettes	89 (40.3)	69 (41.6)	20 (36.4)	
I used to smoke cigarettes	29 (13.1)	23 (13.9)	6 (1.9)	
Godin–Shephard physical activity				.42
Active	66 (29.9)	48 (28.9)	18 (32.7)	
Insufficiently active	79 (35.7)	62 (37.3)	17 (3.9)	
Highest level of education				.28
I have not finished high school	11 (5.0)	10 (6.0)	1 (1.8)	
I have completed high school	21 (9.5)	16 (9.6)	5 (9.1)	
I have not finished college or vocational school	27 (12.2)	23 (13.9)	4 (7.3)	
I finished college or vocational school	62 (28.1)	42 (25.3)	20 (36.4)	
Some/completed graduate or professional school	24 (10.9)	19 (11.4)	5 (9.1)	
Marital status				.07
Single	55 (24.9)	46 (27.7)	9 (16.4)	
Married/partnered	85 (38.5)	59 (35.5)	26 (47.3)	
Divorced/widower	5 (2.3)	5 (3.0)		
Number of antihypertensive medications				.35
0	141 (63.8)	103 (62.0)	38 (69.1)	
1	49 (22.2)	38 (22.9)	11 (20.0)	
2	25 (11.3)	21 (12.7)	4 (7.3)	
3	5 (2.3)	4 (2.4)	1 (1.8)	
≥4	1 (0.5)		1 (1.8)	
Race				.42
Black	49 (22.2)	34 (20.5)	15 (27.3)	
Other	85 (38.5)	63 (38.0)	22 (40.0)	
White	87 (39.4)	69 (41.6)	18 (32.7)	
Self-perceived health status				.21
Excellent	2 (0.9)	1 (0.6)	1 (1.8)	
Very good or good	68 (30.8)	56 (33.7)	12 (21.8)	
Fair	52 (23.5)	36 (21.7)	16 (29.1)	
Poor or no response	22 (10.0)	16 (9.6)	6 (10.9)	
Comorbidities				
Anxiety and/or depression	90 (40.7)	71 (42.8)	19 (34.5)	.28
Chronic kidney disease	1 (0.5)	1 (0.6)	0 (0.0)	.56
Diabetes	11 (5.0)	10 (6.0)	1 (1.8)	.21
Dyslipidemia	23 (10.4)	19 (11.4)	4 (7.3)	.38
Other chronic comorbidity	68 (30.8)	52 (31.3)	16 (29.1)	.76
Risk factors				
E-cigarette/vaping use in past 6 mo	10 (4.5)	9 (5.4)	1 (1.8)	.27
Ever received Medicaid	48 (21.7)	33 (19.9)	15 (27.3)	.25
Family history of heart disease or stroke	52 (23.5)	37 (22.3)	15 (27.3)	.45
Financial status: inadequate income	109 (49.3)	83 (50.0)	26 (47.3)	.73
Detailed 24-h ABPM results				
Ambulatory systolic BP (mm Hg)	131.9 [108.0–169.0]	132.4 [110.0–169.0]	130.1 [108.0–168.0]	.17
Ambulatory diastolic BP (mm Hg)	87.2 [72.0–110.0]	87.8 [78.0–110.0]	85.5 [72.0–106.0]	.06
Daytime systolic ABPM (mm Hg)	135.7 [114.0–173.0]	135.9 [116.0–173.0]	135.1 [114.0–168.0]	.45
Daytime diastolic ABPM (mm Hg)	91.0 [75.0–111.0]	91.1 [76.0–111.0]	90.5 [75.0–110.0]	.46
Night systolic ABPM (mm Hg)	118.9 [0.0–167.0]	119.2 [0.0–164.0]	118.3 [90.0–167.0]	.59
Night diastolic ABPM (mm Hg)	75.5 [0.0–108.0]	76.2 [0.0–108.0]	73.8 [56.0–95.0]	.12
Night dip systolic ABPM (\%)	12.1 [0.0–29.5]	11.7 [0.0–24.3]	13.3 [0.0–29.5]	.47
Night dip diastolic ABPM (\%)	16.9 [0.0–37.2]	15.9 [0.0–33.3]	19.3 [0.0–37.2]	.10
24-h ABPM HTN categorization				
Confirmed chronic HTN	142 (64.3)	108 (65.1)	34 (61.8)	.66
Masked HTN	43 (19.5)	31 (18.7)	12 (21.8)	.61
No confirmed HTN	3 (1.4)	2 (1.2)	1 (1.8)	.73
Nocturnal HTN	56 (25.3)	42 (25.3)	14 (25.5)	.98
White coat HTN	73 (33.0)	54 (32.5)	19 (34.5)	.78

Continuous variables are described using medians [interquartile ranges] and tested using Mann–Whitney–Wilcoxon tests. Categorical variables are described using counts (percentages) and tested using chi-square tests, with the exception of education, number of HTN medications, and self-perceived health status, which were ordered and tested using Mann–Whitney–Wilcoxon tests.

*ABPM*, ambulatory blood pressure monitoring; *BP*, blood pressure; *HTN*, hypertension.

The 2 groups—the excluded group (including individuals with and without a history of pregnancy) and the group with a documented pregnancy history—differed significantly in age, number of children, and race (*P*<.05).

The women with history of pregnancy were older (median [interquartile range], 35.3 [26.0–39.0] vs 32.7 [19.0–39.0] years; *P*=.02) and had more children (0.9 [0.0–6.0] vs 2.3 [0.0–6.0]; *P*<.001). Information on race was collected after enrollment into MyHEART and in the pregnancy history questionnaire. Race was unknown for women who did not enroll. There was no difference in the percentage of Black or White participants between the included and excluded groups (*P*=.85).

Analysis of 24-hour ABPM data showed that among the overall study population (n=221), 73 (33%) had white coat HTN, 56 (25.3%) had nocturnal HTN, and 43 (19.5%) had masked HTN. Of the 55 participants who reported having a prior pregnancy, 19 (34.5%) had white coat HTN, 14 (25.5%) had nocturnal HTN, and 12 (21.8%) had masked HTN. The data comparing women with a prior pregnancy with those without a prior pregnancy are displayed in Table 2. Participants who reported a prior pregnancy were, on average, more likely to be Black and more likely to receive Medicaid compared with those without a prior pregnancy. After excluding participants with unknown race, a higher proportion of White participants reporting a prior pregnancy was observed (*P*=.02).

**Table 2:** Baseline participant characteristics by any prior pregnancy

Variable	Overall	No prior pregnancy	Prior pregnancy	*P* value
Total	125	70	55	
Age, y	33.7 [19.0–39.0]	32.5 [19.0–39.0]	35.3 [26.0–39.0]	.003
Alcohol beverages/wk	2.7 [0.0–23.0]	2.4 [0.0–15.0]	3.1 [0.0–23.0]	.84
Body mass index (kg/m^2^)	34.8 [19.5–61.9]	34.2 [19.5–61.9]	35.5 [21.1–54.3]	.25
Number of children at home	1.1 [0.0–6.0]	0.2 [0.0–2.0]	2.3 [0.0–6.0]	<.001
Office systolic BP (mm Hg)	130.5 [102.0–175.7]	130.7 [102.0–157.0]	130.2 [102.7–175.7]	.57
Office diastolic BP (mm Hg)	88.0 [57.3–130.3]	87.6 [64.7–114.0]	88.4 [57.3–130.3]	.8
Waist circumference (cm)	106.7 [65.0–152.0]	106.0 [65.0–152.0]	107.6 [80.0–145.0]	.70
Weight (kg)	94.0 [53.0–158.0]	92.0 [53.0–158.0]	96.5 [61.0–146.0]	.20
Cigarette tobacco status				.14
I currently smoke cigarettes	13 (10.4)	4 (5.7)	9 (16.4)	
I have never smoked cigarettes	52 (41.6)	32 (45.7)	20 (36.4)	
I used to smoke cigarettes	13 (10.4)	7 (10.0)	6 (10.9)	
Godin–Shephard physical activity				.98
Active	40 (32.0)	22 (31.4)	18 (32.7)	
Insufficiently active	38 (30.4)	21 (30.0)	17 (30.9)	
Highest level of education				.66
I have not finished high school	3 (2.4)	2 (2.9)	1 (1.8)	
I have completed high school	7 (5.6)	2 (2.9)	5 (9.1)	
I have not finished college or vocational school	13 (10.4)	9 (12.9)	4 (7.3)	
I finished college or vocational school	41 (32.8)	21 (30.0)	20 (36.4)	
Some/completed graduate or professional school	14 (11.2)	9 (12.9)	5 (9.1)	
Marital status				
Single	26 (20.8)	17 (24.3)	9 (16.4)	
Married/partnered	52 (41.6)	26 (37.1)	26 (47.3)	
Number of antihypertensive medications				.88
0	87 (69.6)	49 (70.0)	38 (69.1)	
1	25 (20.0)	14 (20.0)	11 (20.0)	
2	10 (8.0)	6 (8.6)	4 (7.3)	
3	2 (1.6)	1 (1.4)	1 (1.8)	
≥4	1 (0.8)		1 (1.8)	
Race				.01
Black	21 (16.8)	6 (8.6)	15 (27.3)	
Other	50 (40.0)	28 (40.0)	22 (40.0)	
White	54 (43.2)	36 (51.4)	18 (32.7)	
Self-perceived health status				.26
Excellent	2 (1.6)	1 (1.4)	1 (1.8)	
Very good or good	35 (28.0)	23 (32.9)	12 (21.8)	
Fair	27 (21.6)	11 (15.7)	16 (29.1)	
Poor or no response	14 (11.2)	8 (11.4)	6 (10.9)	
Comorbidities				
Anxiety and/or depression	48 (38.4)	29 (41.4)	19 (34.5)	.43
Diabetes	4 (3.2)	3 (4.3)	1 (1.8)	.44
Dyslipidemia	14 (11.2)	10 (14.3)	4 (7.3)	.22
Other chronic comorbidity	36 (28.8)	20 (28.6)	16 (29.1)	.95
Risk factors				
E-cigarette/vaping use in past 6 mo	6 (4.8)	5 (7.1)	1 (1.8)	.17
Ever received Medicaid	22 (17.6)	7 (10.0)	15 (27.3)	.01
Family history of heart disease or stroke	28 (22.4)	13 (18.6)	15 (27.3)	.25
Financial status: inadequate income	65 (52.0)	39 (55.7)	26 (47.3)	.35
Detailed 24-h ABPM results				
Ambulatory systolic BP (mm Hg)	131.1 [108.0–168.0]	131.9 [112.0–155.0]	130.1 [108.0–168.0]	.32
Ambulatory diastolic BP (mm Hg)	86.5 [72.0–106.0]	87.3 [78.0–106.0]	85.5 [72.0–106.0]	.18
Daytime systolic ABPM (mm Hg)	135.7 [114.0–168.0]	136.2 [119.0–158.0]	135.1 [114.0–168.0]	.36
Daytime diastolic ABPM (mm Hg)	91.1 [75.0–110.0]	91.6 [80.0–110.0]	90.5 [75.0–110.0]	.34
Night systolic ABPM (mm Hg)	116.4 [0.0–167.0]	114.7 [0.0–150.0]	118.3 [90.0–167.0]	.78
Night diastolic ABPM (mm Hg)	72.9 [0.0–95.0]	72.1 [0.0–94.0]	73.8 [56.0–95.0]	.77
Night dip systolic ABPM (\%)	13.2 [0.0–29.5]	13.0 [0.0–24.3]	13.3 [0.0–29.5]	.97
Night dip diastolic ABPM (\%)	19.0 [0.0–37.2]	18.7 [0.0–33.3]	19.3 [0.0–37.2]	.93
24-h ABPM HTN categorization				
Confirmed chronic HTN	76 (60.8)	42 (60.0)	34 (61.8)	.84
Masked HTN	24 (19.2)	12 (17.1)	12 (21.8)	.51
No confirmed HTN	2 (1.6)	1 (1.4)	1 (1.8)	.86
Nocturnal HTN	31 (24.8)	17 (24.3)	14 (25.5)	.88
White coat HTN	45 (36.0)	26 (37.1)	19 (34.5)	.76

Continuous variables are described using medians [interquartile ranges] and tested using Mann–Whitney–Wilcoxon tests. Categorical variables are described using counts (percentages) and tested using chi-square tests, with the exception of education, number of HTN medications, and self-perceived health status, which were ordered and tested using Mann–Whitney–Wilcoxon tests.

*ABPM*, ambulatory blood pressure monitoring; *BP*, blood pressure; *HTN*, hypertension.

**Figure 2:**
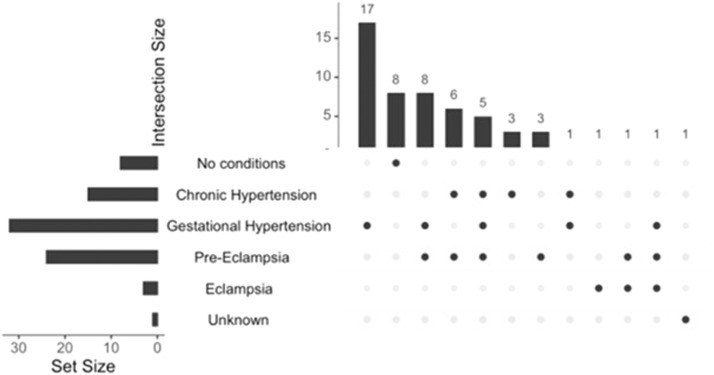
Hypertension types in 55 prior pregnancies

**Figure 3:**
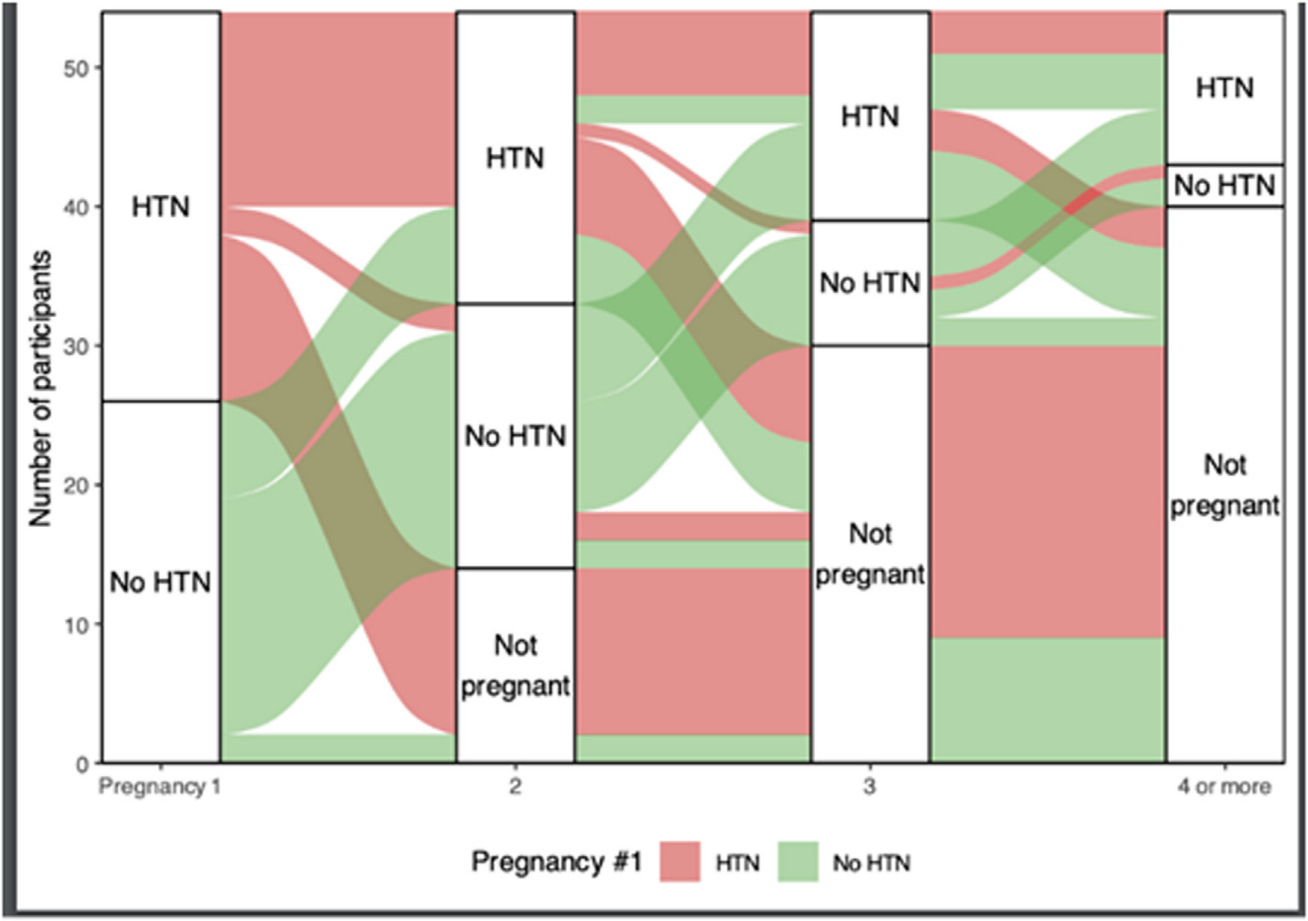
HTN diagnosis patterns across sequential pregnancies *HTN*, hypertension.

Of the 70 women who reported not having a prior pregnancy, 26 (37.1%) had white coat HTN, 17 (24.3%) had nocturnal HTN, and 12 (17.1%) had masked HTN. We further categorized the 55 women according to history of HTN during prior pregnancies. Figure 2 displays the types of hypertensive disorders of pregnancy experienced by participants. Among participants with multiple pregnancies, Figure 3 displays the distribution of HTN diagnoses across sequential pregnancies. Table 3 displays participant characteristics, demographics, and ABPM measurements according to HTN during prior pregnancies. There was no significant statistical difference in any of the assessed values between the 2 groups, except for confirmed HTN/normotensive results by ABPM. Of the 47 participants who reported having some form of HTN during a prior pregnancy, 30 (63.8%) had confirmed HTN, 11 (23.4%) had nocturnal HTN, and 11 (23.4%) had masked HTN.

Gupta. Ambulatory blood pressure monitoring for blood pressure categorization in young women with presumed chronic hypertension. Am J Obstet Gynecol Glob Rep 2026.

Gupta. Ambulatory blood pressure monitoring for blood pressure categorization in young women with presumed chronic hypertension. Am J Obstet Gynecol Glob Rep 2026.

**Table 3:** Baseline participant characteristics by hypertension during prior pregnancy

Variable	Overall	HTN during prior pregnancies	No HTN during prior pregnancies	*P* value
Total	55	47	8	
Age, y	35.3 [26.0–39.0]	35.2 [27.0–39.0]	35.8 [26.0–39.0]	.58
Alcohol beverages/wk	3.1 [0.0–23.0]	3.0 [0.0–23.0]	3.5 [0.0–16.0]	.75
Body mass index (kg/m^2^)	35.5 [21.1–54.3]	35.3 [21.1–54.3]	36.9 [26.2–44.3]	.48
Number of children at home	2.3 [0.0–6.0]	2.4 [1.0–6.0]	1.4 [0.0–4.0]	.14
Office systolic BP (mm Hg)	130.2 [102.7–175.7]	130.6 [102.7–175.7]	127.6 [105.3–158.0]	.74
Office diastolic BP (mm Hg)	88.4 [57.3–130.3]	88.9 [57.3–130.3]	85.5 [70.0–105.3]	.40
Waist circumference (cm)	107.6 [80.0–145.0]	107.3 [80.0–145.0]	109.6 [91.0–123.0]	.59
Weight (kg)	96.5 [61.0–146.0]	96.5 [61.0–146.0]	96.6 [67.0–122.0]	.89
Cigarette tobacco status				.34
I currently smoke cigarettes	9 (16.4)	8 (17.0)	1 (12.5)	
I have never smoked cigarettes	20 (36.4)	18 (38.3)	2 (25.0)	
I used to smoke cigarettes	6 (10.9)	4 (8.5)	2 (25.0)	
Godin–Shephard physical activity				.68
Active	18 (32.7)	15 (31.9)	3 (37.5)	
Insufficiently active	17 (30.9)	15 (31.9)	2 (25.0)	
Highest level of education				.40
I have not finished high school	1 (1.8)	1 (2.1)		
I have completed high school	5 (9.1)	4 (8.5)	1 (12.5)	
I have not finished college or vocational school	4 (7.3)	3 (6.4)	1 (12.5)	
I finished college or vocational school	20 (36.4)	17 (36.2)	3 (37.5)	
Some/completed graduate or professional school	5 (9.1)	5 (10.6)		
Marital status				
Single	9 (16.4)	6 (12.8)	3 (37.5)	
Married/partnered	26 (47.3)	24 (51.1)	2 (25.0)	
Number of antihypertensive medications				.74
0	38 (69.1)	32 (68.1)	6 (75.0)	
1	11 (20.0)	10 (21.3)	1 (12.5)	
2	4 (7.3)	3 (6.4)	1 (12.5)	
3	1 (1.8)	1 (2.1)		
≥4	1 (1.8)	1 (2.1)		
Race				.41
Black	15 (27.3)	12 (25.5)	3 (37.5)	
Other	22 (40.0)	18 (38.3)	4 (50.0)	
White	18 (32.7)	17 (36.2)	1 (12.5)	
Self-perceived health status				.59
Excellent	1 (1.8)	1 (2.1)		
Very good or good	12 (21.8)	10 (21.3)	2 (25.0)	
Fair	16 (29.1)	13 (27.7)	3 (37.5)	
Poor or no response	6 (10.9)	6 (12.8)		
Comorbidities				
Anxiety and/or depression	19 (34.5)	16 (34.0)	3 (37.5)	.85
Diabetes	1 (1.8)	1 (2.1)	0 (0.0)	.68
Dyslipidemia	4 (7.3)	4 (8.5)	0 (0.0)	.39
Other chronic comorbidity	16 (29.1)	13 (27.7)	3 (37.5)	.57
Risk factors				
E-cigarette/vaping use in past 6 mo	1 (1.8)	1 (2.1)	0 (0.0)	.68
Ever received Medicaid	15 (27.3)	14 (29.8)	1 (12.5)	.31
Family history of heart disease or stroke	15 (27.3)	13 (27.7)	2 (25.0)	.88
Financial status: inadequate income	26 (47.3)	24 (51.1)	2 (25.0)	.17
Detailed 24-h ABPM results				
Ambulatory systolic BP (mm Hg)	130.1 [108.0–168.0]	130.5 [108.0–168.0]	127.6 [111.0–135.0]	.98
Ambulatory diastolic BP (mm Hg)	85.5 [72.0–106.0]	86.0 [72.0–106.0]	82.6 [72.0–92.0]	.49
Daytime systolic ABPM (mm Hg)	135.1 [114.0–168.0]	135.4 [114.0–168.0]	133.0 [122.0–140.0]	.95
Daytime diastolic ABPM (mm Hg)	90.5 [75.0–110.0]	91.1 [75.0–110.0]	86.8 [76.0–94.0]	.48
Night systolic ABPM (mm Hg)	118.3 [90.0–167.0]	118.9 [90.0–167.0]	115.2 [92.0–126.0]	.98
Night diastolic ABPM (mm Hg)	73.8 [56.0–95.0]	74.1 [56.0–95.0]	71.8 [62.0–83.0]	.69
Night dip systolic ABPM (\%)	13.3 [0.0–29.5]	13.3 [0.0–29.5]	13.3 [5.6–25.3]	.96
Night dip diastolic ABPM (\%)	19.3 [0.0–37.2]	19.8 [0.0–37.2]	16.9 [6.2–29.7]	.58
24-h ABPM HTN categorization				
Confirmed chronic HTN	34 (61.8)	30 (63.8)	4 (50.0)	.46
Masked HTN	12 (21.8)	11 (23.4)	1 (12.5)	.49
No confirmed HTN	1 (1.8)	0 (0.0)	1 (12.5)	.01
Nocturnal HTN	14 (25.5)	11 (23.4)	3 (37.5)	.40
White coat HTN	19 (34.5)	16 (34.0)	3 (37.5)	.85

Continuous variables are described using medians [interquartile ranges] and tested using Mann–Whitney–Wilcoxon tests. Categorical variables are described using counts (percentages) and tested using chi-square tests, with the exception of education, number of HTN medications, and self-perceived health status, which were ordered and tested using Mann–Whitney–Wilcoxon tests.

*ABPM*, ambulatory blood pressure monitoring; *BP*, blood pressure; *HTN*, hypertension.

We analyzed data according to race among the 55 women included in the study (Table 4). Analysis showed that 14 of the 19 (74%) Black participants as opposed to 20 of the 36 (56%) White participants had confirmed HTN. Black women had significantly higher ambulatory systolic BP readings (136.53 vs 125.48; *P*=.017) and an increased incidence of nocturnal HTN compared with their White counterparts (53% vs 11%; *P*<.001).

**Table 4:** Ambulatory blood pressure monitoring results and hypertension types diagnosed for female participants with prior pregnancy, categorized by race

Type	Black	White	*P* value
Total	19	36	
Office systolic BP (mm Hg)	135.05 (18.82)	127.56 (15.21)	.19
Office diastolic BP (mm Hg)	94.21 (14.20)	85.41 (10.76)	.04
Ambulatory systolic BP (mm Hg)	136.53 (15.17)	125.48 (10.78)	.02
Ambulatory diastolic BP (mm Hg)	86.33 (10.20)	84.95 (6.76)	.7
White coat HTN	4/19 (21%)	15/36 (42%)	.13
Nocturnal HTN	10/19 (53%)	4/36 (11%)	<.001
Masked HTN	5/19 (26%)	7/36 (19%)	.56
No confirmed HTN	0/19 (0%)	1/36 (3%)	.46
Confirmed chronic HTN	14/19 (74%)	20/36 (56%)	.19

*BP*, blood pressure; *HTN*, hypertension.

## Discussion

### Principal findings

Among the overall population, we observed that only 64.3% of the women had a presumed diagnosis of chronic HTN, underscoring the inaccuracy of HTN categorization in women of childbearing age. Among participants with a reported history of pregnancy, 63.8% had confirmed chronic HTN, raising concern for mismanagement of HTN during pregnancy. The 24-hour ABPM systolic and diastolic measurements were significantly higher for Black participants than White participants. Furthermore, the prevalence of nocturnal HTN was also higher among Black participants.

As illustrated in Figure 3, we also observed that women who experienced HTN during their first pregnancy were less likely to have subsequent pregnancies compared with those who did not have HTN during their first pregnancies. Among women who had additional pregnancies, the percentage who experienced a hypertensive disorder of pregnancy increased with each subsequent pregnancy.

### Results

The clinical significance of white coat, masked, and nocturnal HTN has been increasingly recognized in the literature, particularly their association with adverse cardiovascular outcomes. However, there is a lack of consensus on standardized diagnostic criteria and monitoring strategies for the different hypertensive categorizations in pregnancy. Multiple studies have demonstrated that ABPM improves risk stratification by identifying these subtypes, which are underdiagnosed with office-only BP measurements. Despite growing evidence, most of these data are derived from older or mixed-sex populations, with limited research focused specifically on women of reproductive age.^7^^,^^8^^,^^10^^,^^12^^,^^14^^,^^19^^,^^20^

Both ACOG, in its Practice Bulletin on hypertension in pregnancy, and the AHA recommend considering out-of-office BP monitoring, such as ABPM or HBPM, to confirm the diagnosis of HTN.^4^^,^^16^ However, in practice, most current guidelines continue to rely on office BP measurements for diagnosis and management. Moreover, there is a scarcity of ABPM devices validated specifically for use in pregnancy, due to physiological changes that may affect measurement accuracy.^9^^,^^16^

### Clinical implications

Miscategorization of HTN and the implications of resultant potential over- or undertreatment can have detrimental effects on the health of childbearing women. White coat HTN has been associated with increased risk for the development of sustained HTN, target organ damage, preterm birth, and cardiovascular events.^8^^,^^21^ Women diagnosed with white coat HTN before 20 weeks of pregnancy showed a 5-fold greater risk of preeclampsia compared with normotensive women.^13^ Although there are no established recommendations for the treatment of white coat HTN, its identification warrants a different clinical approach, which includes close monitoring and potential lifestyle modification to prevent progression to sustained HTN. Early diagnosis is crucial because delaying intervention can increase the risk of cardiovascular damage. The International Society for the Study of Hypertension in Pregnancy (ISSHP) and AHA have proposed that all patients with clinic BP of ≥140/90 mm Hg before 20 weeks of pregnancy undergo ABPM or home blood pressure monitoring (HBPM) to confirm a diagnosis of chronic HTN and rule out white coat HTN.^13^^,^^16^^,^^22^ If white coat HTN is confirmed, proper management of this diagnosis could optimize pregnancy outcomes. In women with white coat HTN, antihypertensive medication management is not recommended; rather, close monitoring with home BP measurements throughout pregnancy for the development of preeclampsia is warranted.^13^ Although HBPM is a safe and acceptable method for cardiovascular surveillance and can allow earlier identification of HTN, prior clinical trials assessing the use of HBPM in pregnancy did not show reductions in maternal or perinatal clinical outcomes.^23^^,^^24^ Thus, HBPM should be used as an adjunct rather than replacement for ABPM, which is required for detection and exclusion of nocturnal HTN. Although the literature is lacking in evidence that ABMP can improve perinatal outcomes, it does document increased detection of preeclampsia, which could therefore provide useful clinical data points to guide pregnancy care.^16^^,^^25^

Masked HTN presents a different challenge. Often not detected and thus undertreated, it is associated with increased risk of cardiovascular events, target organ damage, and mortality—risks comparable to chronic HTN.^26^ Although identifying masked HTN in the general population would require universal ABPM testing, which is not feasible, the clinical context in this study is distinct. This condition underscores the need for comprehensive BP assessment, such as ABPM, in patients with a known diagnosis of chronic HTN to assess BP beyond office measurements. Specifically, in the context of this study, subsequent normal clinic BPs in women with known chronic HTN may be interpreted as adequate control or may raise uncertainty regarding the persistence of the diagnosis itself. In this setting, ABPM does not serve as a population-wide screening tool, but rather as an extension of surveillance to determine whether normotension in clinic reflects true BP control or masked HTN outside the clinical environment. Consistent with this targeted approach, in the overall (male and female) cohort of the original trial, 39% of male and female participants had ABPM results consistent with masked HTN, highlighting the potential for missed disease if assessment relies solely on office measurements. In this subanalysis of only female participants regardless of pregnancy status, masked HTN was slightly less common than in male participants but still present in 19.5% of female participants with a presumed diagnosis of chronic HTN. Failure to recognize and treat masked HTN represents a missed opportunity for timely intervention and prevention of cardiovascular complications.

Nocturnal HTN is another underrecognized subtype that has been linked to end-organ damage, including left ventricular hypertrophy, silent cerebral disease, and early-onset preeclampsia. In fact, even when office and daytime BPs are well-controlled, isolated nocturnal HTN remains an independent risk factor for cardiovascular disease. This highlights the necessity of monitoring nighttime BP, which requires a unique treatment approach, especially regarding timing and titration of antihypertensive therapy.^12^^,^^19^^,^^20^^,^^27^

These diagnostic challenges are particularly consequential in women of childbearing age, for whom miscategorization can directly affect pregnancy outcomes. For example, women diagnosed with chronic HTN are at higher risk of developing preeclampsia, and both the United States Preventive Services Task Force and ACOG recommend initiating aspirin for its prevention.^16^^,^^28^ During pregnancy, antihypertensive medications are prescribed as standard of care if BP exceeds 140/90 mm Hg. As demonstrated in the CHAP (Chronic Hypertension and Pregnancy) trial, with adequate BP control, there is a decrease in the composite pregnancy outcome of preterm birth, severe preeclampsia, placental abruption, and fetal death.^29^ However, if chronic HTN is not recognized, patients may be diagnosed with preeclampsia rather than exacerbation of chronic HTN, for which BP management differs; antihypertensive medications are not initiated until severe-range BPs occur (>160/110 mm Hg),^30^ potentially requiring prolonged hospitalization and/or preterm delivery. Conversely, women misclassified as having chronic HTN when they have white coat HTN may be incorrectly diagnosed with preeclampsia. In people with known white coat HTN, the AHA proposes management including HBPM; if BPs are normal outside the clinic, preeclampsia is unlikely and pregnancy may be allowed to progress to later gestation, avoiding unnecessary morbidity due to overdiagnosis of preeclampsia.^13^ Therefore, improved BP evaluation and follow-up are especially important during pregnancy, specifically for patients with known white coat HTN. BP management and targets during pregnancy differ from those outside pregnancy, and individualized evaluation and accurate HTN classification provide an opportunity to directly improve pregnancy outcomes.^9^

Our data also reveal significant racial disparities in HTN outcomes among women with a history of pregnancy, which are consistent with the literature. Black women tend to have higher office BP measurements and a greater prevalence of nocturnal HTN compared with White women.^31^^,^^32^ These patterns are essential to address because they contribute to Black women experiencing the highest burden of cardiovascular disease compared with women of other racial groups, including higher risks of pregnancy complications and long-term cardiovascular complications for mothers and their offspring.^32^

Therefore, the use of ABPM is of vital importance for Black women, especially those entering pregnancy, because it enables detection of nocturnal HTN and appropriate classification of HTN. However, it is important to note that addressing these disparities warrants more than improved diagnostic strategies. Efforts must also focus on recognizing and addressing the structural, environmental, and socioeconomic factors, including barriers to healthcare access and food insecurity, which have created and continue to perpetuate these inequities.

### Research implications

ABPM remains the gold standard for diagnosing HTN.^4^ The results from this study reinforce its utility in confirming the diagnosis of chronic HTN, particularly during pregnancy. This is especially relevant for Black women, among whom the prevalence of HTN is disproportionately higher and office-based measurements alone may underestimate the disease burden. Nevertheless, important limitations to ABPM exist, including limited access, increased cost, and decreased feasibility. Moreover, research is needed to validate the use of ABPM devices specifically during pregnancy and to develop strategies that improve equitable access to this diagnostic tool.

### Strengths and limitations

This study has several notable strengths. It is a planned secondary analysis of a randomized controlled trial. All 24-hour ABPM was conducted within a controlled environment (the clinical research office) and performed per protocol using US Food and Drug Administration–approved,^33^ validated devices, with application and interpretation by trained personnel, enhancing internal validity and minimizing risk of bias. Participants were instructed to carry out their usual day-to-day activities during the monitoring. The monitoring protocol included frequent measurements during both wake and sleep periods, capturing circadian BP patterns and enabling assessment of different HTN categorizations.

The study included a large cohort of young adult women, a demographic for whom BP data are seldom available. The large sample size allowed for robust subgroup analyses, including stratification by race and pregnancy history. Given limited availability of ABPM in clinical practice, access to large-volume 24-hour ABPM data is rare in clinical research. The study design and unique population address key gaps in the literature and provide findings that have implications for improving HTN diagnosis and management in at-risk populations.

Several limitations exist. There is potential for recall bias given that all pregnancy-related data were self-reported. The response rates were low, limiting pregnancy history collection to 96 participants. Eligibility criteria for the broader MyHEART study did not assess or exclude individuals with gestational HTN as their sole diagnosis, which may have inadvertently inflated the eligible cohort. Although the overall sample size was substantial, it may still have been underpowered to detect smaller effect sizes, potentially limiting the study’s power.

**Figure 4:**
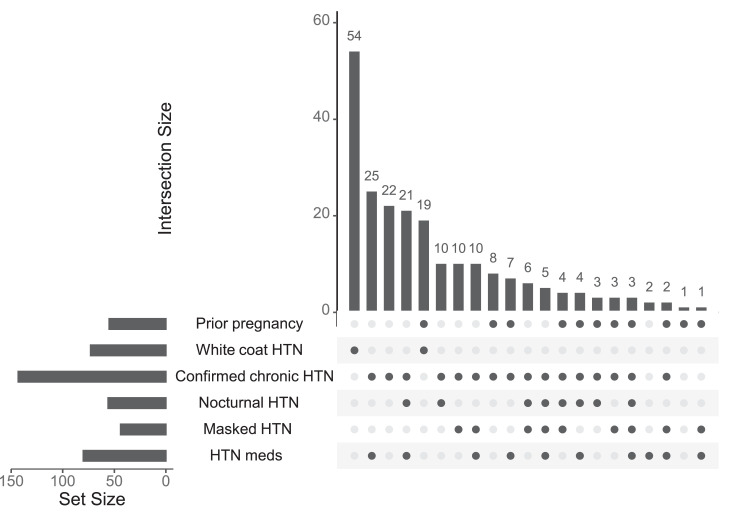
HTN classification by ABPM in 55 previously pregnant women *ABPM*, ambulatory blood pressure monitoring; *HTN*, hypertension.

Although data on participants’ HTN status immediately before or after pregnancy, the timing of their most recent pregnancy relative to ABPM, and their history of multiple pregnancies were not collected, these limitations do not substantially detract from the clinical applicability of the findings. In cardiology and primary care settings, 24-hour ABPM is routinely used to confirm chronic HTN, evaluate adequacy of BP control, and detect nocturnal, masked, or white coat HTN in young adults with office-based diagnoses. The fact that 36.2% (80/221) of participants were receiving antihypertensive therapy during ABPM reflects common real-world practice, in which patients frequently present already on medication. For the MyHEART trial, if participants were prescribed and taking antihypertensive therapy and were normotensive, they were deemed well controlled and therefore ineligible for the main trial.^18^ Figure 4 shows the ABPM results defining HTN classification, considering antihypertensive status. All participants diagnosed with white coat HTN were not receiving antihypertensive therapy. ABPM remains informative even in the remaining cohort, including those prescribed antihypertensive therapy, because it allows clinicians to distinguish true treatment control from residual uncontrolled HTN and to reassess the accuracy of initial diagnoses. Thus, despite these methodological constraints, ABPM continues to provide clinically meaningful diagnostic and management insights.

Gupta. Ambulatory blood pressure monitoring for blood pressure categorization in young women with presumed chronic hypertension. Am J Obstet Gynecol Glob Rep 2026.

Lastly, the analysis did not correct for multiple comparisons, increasing the risk of type I errors.

### Conclusions

Our analysis underscores that many women of childbearing age, both with and without prior pregnancies, lack an accurate diagnosis of HTN. Misclassification in this population carries significant risk because both over- and undertreatment can adversely influence pregnancy-related outcomes, including medication selection, timing and mode of delivery planning, and broader management decisions that affect maternal and fetal health. Incorporating ABPM and differentiating among chronic, white coat, masked, and nocturnal HTN are therefore essential to improving diagnostic precision. Accurate phenotyping enables informed treatment decisions, targeted lifestyle modifications, and care aligned with true cardiovascular risk profiles, thereby optimizing health outcomes.

## CRediT authorship contribution statement

**Srishti Gupta:** Writing – review & editing, Writing – original draft, Methodology, Conceptualization. **Megan R. Knutson Sinaise:** Writing – review & editing, Writing – original draft, Project administration, Data curation. **Jennifer Zaborek:** Writing – review & editing, Writing – original draft, Formal analysis. **Kara K. Hoppe:** Writing – review & editing, Writing – original draft, Supervision, Methodology, Investigation, Funding acquisition, Conceptualization.

## Appendix Phone Script

### Diagnosis of hypertension

1. Had you been diagnosed with high blood pressure by a medical provider prior to your first two MyHEART study appointments?

### Pregnancy history

2. Had you experienced pregnancy at any time prior to your first two MyHEART Study appointments?
a. If no, end call.
3. Number of pregnancies and gestational age of baby at delivery (rounded to week) up until your first MyHEART Study appointments?
4. Number of fetuses in all your past pregnancies?

### Hypertension before pregnancy

5. Do you currently take blood pressure medication?
6. Had you ever been told you have white coat hypertension before your first MyHEART appointments?
7. Did you have high blood pressure before any pregnancies, confirmed diagnosis by a medical provider (1st pregnancy if multiple pregnancies), before your first MyHEART Study appointments?

### Hypertension during/after pregnancy

8. Were you diagnosed with chronic hypertension after pregnancy/post-partum?
9. Did you have high blood pressure during pregnancy? If no, skip to question 21.
10. If you had high blood pressure during pregnancy, what type of hypertension was it?
11. If you had high blood pressure during pregnancy, did you take medications to control your blood pressure during your pregnancy?
12. If you had high blood pressure during pregnancy, did you take medications to control your blood pressure after pregnancy through 6 weeks post-partum?
13. If you had high blood pressure during pregnancy, did you take medications to control your blood pressure beyond 6 weeks post-partum?

### Health of baby(ies)

14. Did your baby(ies) have any complications at/after delivery?

### Demographics

15. What is your race?
16. What was your marital status at the time of your first two MyHEART Study appointments?
17. What was your highest level of education at the time of your first two MyHEART Study appointments?
